# Clinical Findings, Follow-up and Treatment Results in Patients with Ocular Rosacea

**DOI:** 10.4274/tjo.48902

**Published:** 2016-01-05

**Authors:** İlkay Kılıç Müftüoğlu, Yonca Aydın Akova

**Affiliations:** 1 İstanbul Training and Research Hospital, Ophthalmology Clinic, İstanbul, Turkey; 2 Bayındır Kavaklıdere Hospital, Ophthalmology Clinic, Ankara, Turkey

**Keywords:** Ocular rosacea, treatment, complications, cyclosporine

## Abstract

**Objectives:**

To report the clinical features, treatment options and complications in patients with ocular rosacea.

**Materials and Methods:**

The records of 48 eyes of 24 patients with ocular rosacea were retrospectively reviewed. Patients’ ocular signs and symptoms were scored between 1 and 4 points according to disease severity; tear film break-up time (BUT) and Schirmer’s test results were recorded before and after the treatment. Preservative-free artificial tears, topical antibiotic eye drops/ointments, short-term topical corticosteroids, topical 0.05% cyclosporine and oral doxycycline treatment were applied as a standard therapy to all patients. Additional treatments were given as needed. Complications were recorded.

**Results:**

Twenty-four patients with a mean age of 48.5±35.4 (32-54) years were followed for a mean 15±9.4 (8-36) months. Ocular findings included meibomitis in 100% of cases, anterior blepharitis in 83% (40 eyes), punctate keratopathy in 67% (32 eyes), chalazia in 50% (24 eyes), corneal neovascularization in 50% (24 eyes) and subepithelial infiltrates in 16.6% (8 eyes). Significant improvement of symptoms and clinical findings were achieved in all patients with treatment. The increases in Schirmer’s test and BUT were 3.3±1.5 and 4.5±2.8, respectively (p<0.05). Descemetocele and small corneal perforation occurred in 2 eyes; re-epithelialization was achieved in both eyes with tissue adhesive application (1 eye) and additional amniotic membrane transplantation (1 eye). Four eyes of three patients showed significant regression of corneal neovascularization with topical bevacizumab therapy.

**Conclusion:**

Ocular rosacea may present with a variety of ophthalmic signs. It is possible to control the ophthalmic disease with appropriate therapeutic modalities including topical corticosteroids, topical cyclosporine and systemic doxycycline.

## INTRODUCTION

Rosacea is a chronic skin condition affecting the blood vessels and sebaceous glands and is characterized by recurrent erythema, telangiectasia, papules and pustules.^1,2,3^ Although usually presenting with skin involvement, ocular involvement is found in 58-72% of patients with acne rosacea.^1^ In approximately 20% of patients, ocular signs appear prior to dermatological signs.^1,2^ Signs of ocular rosacea include anterior blepharitis, meibomitis, recurrent chalazia, eyelid erythema and telangiectasia, interpalpebral conjunctival hyperemia, and peripheral corneal vascularization.^1,4,5^ Complications in cases with severe corneal involvement can also lead to vision loss.^5,6^

Though the etiology of rosacea is not fully understood, immune system dysfunction, inflammatory reaction to cutaneous microorganisms, Demodex folliculorum infestation, environmental factors like sunlight, and vascular anomalies have been implicated in its pathogenesis.^2,3^ By inducing changes at the vascular level, environmental factors such as sunlight and temperature changes are claimed to lead to vascular dilation, increased capillary permeability and edema, which create an ideal environment for the proliferation of Demodex folliculorum.^2,3,6^

Furthermore, it has been shown that proinflammatory cytokine release is triggered in these patients due to immune system dysfunction.^7^ This hypothesis is supported by findings of significantly elevated levels of proinflammatory cytokines (interleukin-1 alpha) and the degradative enzyme matrix metalloproteinase-9 in patients’ tears, and the subsequent improvement of symptoms after inhibiting these factors.^7,8^

Rosacea patients show increased bacterial lipase activity in their meibomian gland secretions due to increased bacterial flora on the eyelids. This causes the release of toxic free fatty acids, which contribute to the pathogenesis of the disease.^2,7^

There are several different approaches and treatment choices available for the treatment of rosacea,^5,6,7,8,9,10^ including lubricating agents, topical steroids, topical antibiotic eye drops and ointments, and systemic antibiotics. As these patients often have concomitant dry eye syndrome, dry eye therapy may also be necessary.^9^

In this study, we aimed to report the clinical characteristics, treatment options and complications of ocular rosacea.

## MATERIALS AND METHODS

The records of patients who were followed in the Başkent University Faculty of Medicine and the Ophthalmology Clinic of the Bayındır Kavaklıdere Hospital for ocular rosacea between January 2008 and December 2013 were retrospectively analyzed.

Diagnosis was based on the presence of anterior blepharitis, meibomitis and/or meibomian gland dysfunction (MGD), recurrent chalazion, eyelid telangiectasia, punctate epitheliopathy, corneal infiltrates or neovascularization, particularly in the peripheral cornea. Patients who first presented to the ophthalmology clinic but also exhibited dermatological symptoms were given an initial diagnosis of rosacea and the dermatology clinic was consulted to confirm the diagnosis. Patients who presented to the dermatology clinic due to skin symptoms and were diagnosed with acne rosacea were evaluated for ocular involvement in our clinic. Patients received topical metronidazole prescribed by the dermatology clinic and our standard ophthalmologic treatment regimen, with no additional systemic treatments.

At each visit, all patients underwent a detailed ophthalmologic examination including best corrected visual acuity measurement, slit-lamp anterior segment examination, and tear film break-up time (BUT) and Schirmer’s tests. Patients’ signs and symptoms were scored on a scale of 0-4 points based on their severity before treatment and at their last follow-up visit. Table 1 shows the scoring scale of ocular symptoms; Table 2 shows the scoring scale of ocular findings.

All patients were treated with hot compresses, eyelash base cleansing, preservative-free artificial tear drops (Tears Naturale Free, Alcon, Fort Worth, Texas, USA or Refresh, Allergan Inc, Irvine, CA, USA), artificial tear gel (Thilotears SE, Liba Lab., İstanbul, Turkey), topical antibiotic ointment (Ciloxan ophthalmic ointment, Alcon), short-term low-dose topical corticosteroid drops (loteprednol etabonate or fluorometholone) four times daily, 100 mg oral doxycycline once daily, and topical 0.05% cyclosporine drops (Restasis, Allergan) four times daily. Fluorometholone (FML, Allergan) was used as the topical corticosteroid drops between January 2008 and December 2010, and loteprednol etabonate drops (Lotemax, Bausch&Lomb, Bridgewater, NJ, USA) were used after December 2010. Additional medical and/or surgical treatments applied when necessary and complications were recorded.

Patients with structural abnormalities of the eyelids, aqueous-deficient dry eye, inflammatory or infectious keratitis, or previous intraocular surgery were not included in the study.

The Wilcoxon signed rank test was used to evaluate the patients’ pre- and post-treatment signs and symptoms due to their nonparametric values.

## RESULTS

Forty-eight eyes of 24 patients (18 women, 6 men) with a mean age of 48.5±35.4 (32-54) years were included in the study. The mean follow-up time was 15±9.4 (8-36) months.

**Figure 1:**
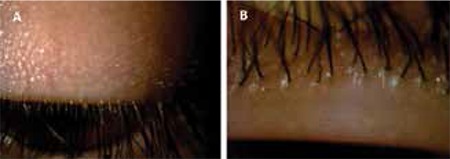
Anterior (A) and posterior (B) signs of blepharitis

**Figure 2:**
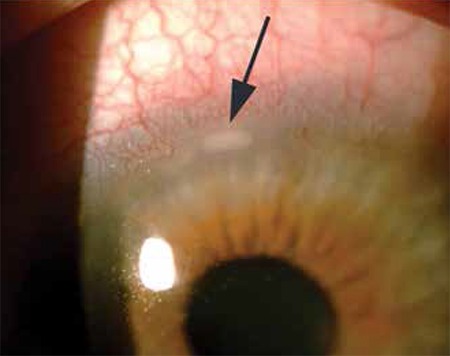
Anterior segment photograph showing peripheral sterile corneal infiltrate

**Figure 3:**
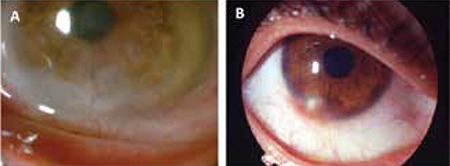
Anterior segment photographs showing corneal neovascularization (A) and peripheral corneal infiltrate (B)

**Figure 4:**
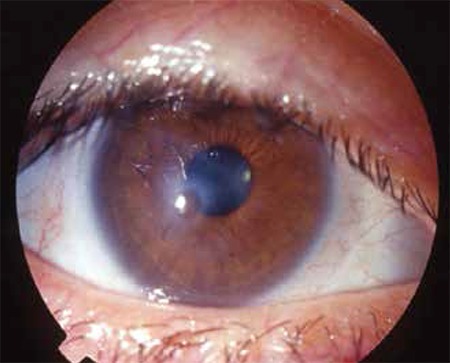
Anterior segment photograph of an ocular rosacea patient with descemetocele

At the time of diagnosis, meibomitis/MGD was present in all eyes (100%), anterior blepharitis in 40 eyes (83.3%), punctate keratopathy in 32 eyes (67%), chalazion in 24 eyes (50%), corneal neovascularization in 24 eyes (50%), and peripheral subepithelial infiltrates in 8 eyes (16.6%). Figure 1 and Figure 2 show anterior segment photographs of patients with anterior and posterior blepharitis, and peripheral sterile corneal infiltrates, respectively. Figure 3 and Figure 4 show anterior segment photographs of patients with corneal neovascularization and peripheral corneal infiltrates, and descemetocele, respectively.

Hot compresses and massage were recommended to all patients as initial treatment. All patients were administered preservative-free artificial tears four times daily; artificial tear gel once daily if necessary; topical corticosteroid eye drops four times daily, decreasing by one drop each week for four weeks; topical antibiotic ointment for 10 days; 100 mg/day systemic doxycycline once daily for a mean of 6.8±2.1 (4-13) months; and 0.05% topical cyclosporine eye drops four times daily for a mean 9.7±3.0 (5-13) months. Topical corticosteroid eye drops were reintroduced in two patients who had exacerbations of their signs and symptoms in the third and fourth months of treatment. Their corticosteroid treatment consisted of four drops daily decreasing by one drop each week for four weeks, followed by one drop every other day for a week and one drop every third day for another week. Peripheral sterile infiltrates were observed during follow-up in 8 eyes of 8 patients; these eyes were treated with topical steroid eye drops starting four times daily and gradually decreased according to clinical response to treatment.

All patients showed significant improvement in their ocular signs and symptoms at the end of the follow-up period. Compared to pre-treatment values, Schirmer’s scores increased 3.3±1.5 mm and BUT increased 4.5±2.8 s (p<0.05, p<0.05). Patients’ mean pre- and post-treatment symptoms scores are shown in Table 3.

Four eyes of 3 patients that did not show sufficient clinical response and/or developed severely sight-threatening choroidal neovascularization despite standard therapies were treated with 5 mg/ml topical bevacizumab eye drops four times daily. The topical bevacizumab treatment was discontinued in each of the 4 eyes when clinical remission of the corneal neovascularization was observed (after 3, 3.5, 4 and 5 months in the four eyes).

Two patients presented to our clinic with descemetocele formation and corneal perforation; their histories revealed that they had been diagnosed with ocular rosacea and followed at a different center. Corneal re-epithelization was achieved with tissue adhesive and bandage contact lens in one of these patients and with tissue adhesive and amniotic membrane transplantation in the other. After re-epithelization the patients were followed up with the standard treatments (12 months for one patient, 14 for the other). At final examination both patients were stable.

None of the patients developed complications related to the use of steroid, cyclosporine and/or topical bevacizumab eye drops during the follow-up period.

All patients showed improvement of their ocular signs and symptoms at the end of the follow-up period, and recurrence was not observed in any of the patients during follow-up. Ten patients used artificial tears for maintenance therapy as needed.

## DISCUSSION

Ocular rosacea may present with various ocular signs.^5,6^ However, it is still not entirely clear which treatment approaches are effective.

Mild or moderate ocular rosacea can be treated with hot compresses, baby shampoo for lid hygiene and artificial tears, while a more intense lubricant in the form of a gel or ointment can be used for long-term comfort.^11,12^

Although there are studies showing decreased local inflammation of the meibomian glands with a combination of lid hygiene and topical antibiotic application alone, these agents do not have the desired effect on the ocular surface.^11,12^ Therefore we utilize short-term topical steroid and long-term topical cyclosporine and oral doxycycline as adjunct therapies to the standard treatment of lid hygiene and artificial tears. With this treatment we achieved significant improvement in our patients’ ocular signs and symptoms.

Doxycycline administered orally to treat rosacea has both antibacterial properties as well as an anti-inflammatory effect. Doxycycline inhibits neutrophil chemotaxis, angiogenesis, lymphocyte proliferation and blocks matrix metalloproteinase activity as well as collagenase and lipase production.^10,11,12,13,14,15^ Thus, as in cutaneous rosacea, it has also been successfully used as an adjunct to topical therapies in ocular rosacea.^10,11,12,13,14,15^ Compared to other tetracyclines, doxycycline has fewer side effects. In a study including 15 ocular rosacea patients, slow-release doxycycline at a dose of 40 mg once daily resulted in a marked decrease in ocular complaints in 80% of patients.^8^ However, studies including larger numbers of patients are necessary to determine the efficacy of doxycycline at low doses. It should also be kept in mind that many patients experience exacerbations when tetracycline use is discontinued. Thus, we prescribed doxycycline for a mean duration of 6 months, and we believe doxycycline also contributed to the clinical improvements observed in our patients. However, long-term use of tetracyclines can result in gastrointestinal system intolerance, which may cause a drug compliance problem and necessitate discontinuation of the drug. Recent studies have shown that topical azithromycin is also effective in the treatment of posterior blepharitis.^16,17^

Episcleritis, scleritis, iritis and sterile keratitis may occur subsequently to persistent ocular inflammation. Nine eyes of 8 patients in our series exhibited sterile peripheral corneal infiltrates. Steroid eye drop treatment was reintroduced in these patients, all of whom showed significant or complete regression of the infiltrates. Because the long-term use of steroids has potential side effects like cataract and glaucoma, they are recommended for acute exacerbations and cases of sterile infiltrates, applied in gradually tapering doses. We did not see any side effects related to topical steroid use in our study.

The pathophysiology of rosacea is still not fully understood, but there are arguments pointing toward the dysregulation of inflammatory and vascular responses to immune agents.^7^ Considering the anti-inflammatory effect of cyclosporine A and its efficacy in the treatment of meibomitis, we also prescribed 0.05% topical cyclosporine four times daily as an adjunct therapy. Significant improvement in ocular signs and symptoms was observed with this treatment in particular. Ocular surface inflammation was controlled, and we observed longer BUT and higher Schirmer’s test values. These effects were likely due to 0.05% topical cyclosporine inhibiting active lymphocytes in the conjunctiva, thereby decreasing inflammation and increasing tear production. Schechter et al.^9^ followed 34 patients with ocular rosacea and found that patients treated with 0.05% topical cyclosporine twice daily had longer BUT, higher Schirmer’s test values and corneal staining improvement compared to patients who used artificial tears. These effects suggest that topical cyclosporine has utility in the treatment of ocular rosacea. Cyclosporine is usually applied twice daily in mild cases of dry eye, but more frequent application has been shown to result in greater improvement of ocular signs and symptoms in more severe cases.^15,18,19^ We found that topical cyclosporine applied four times daily was well tolerated and effective. Furthermore, the safe anti-inflammatory profile of topical cyclosporine allows it to be used in the long term and while the disease is in remission. The results of our study indicate that topical cyclosporine use in combination with other treatments provides important benefits in the management of ocular rosacea.

Corneal neovascularization, especially in the inferior quadrant, occurs with ocular rosacea. Vessels advancing into the cornea cause corneal inflammation, scarring, edema and lipid accumulation, which can lead to reduction in corneal transparency and impaired vision; therefore, the treatment of these patients is very important. Although systemic antibiotics, cyclosporine and steroids can be applied in corneal neovascularization, the increasing use and reported efficacy of anti-vascular endothelial growth factor (VEGF) agents in the treatment of retinochoroidal diseases has brought these agents to the fore in the prevention of corneal neovascularization.^20,21,22,23,24^ Bevacizumab inhibits vascular endothelial cell migration and proliferation and decreases vascular permeability by blocking VEGF-A.^23,24^ Local or topical bevacizumab can be successfully used to treat ocular surface neovascularization when other therapies have been insufficient or to enhance their efficacy.^21,22,23,24^ Cheng et al.^25^ treated corneal neovascularization with 3 weeks of 1.0% topical bevacizumab and followed the patients for 24 weeks; they found reductions in the area of neovascularization in week 6 and in vessel diameter in week 12. Koenig et al.^26^ observed new epithelial defects in 16.7% of their cases and spontaneous corneal perforation in one case after topical bevacizumab treatment. In our study, 4 eyes that developed corneal neovascularization due to ocular rosacea were treated with topical bevacizumab with the primary aim of stabilizing the ocular surface and to avoiding aggravation of corneal epitheliopathy. We started topical bevacizumab treatment 4 weeks after initial presentation in 3 cases and 5 weeks after in 1 case, and we observed significant clinical reduction of the neovascularization with the application of 5 mg/ml 4 times daily. According to our results, we believe that topical bevacizumab may be an effective adjunct therapy in rosacea-related ocular surface neovascularization when required.

The most severe sight-threatening complications of ocular rosacea are stromal thinning and corneal perforation.^27,28,29^ The underlying cause of corneal thinning has not been clearly determined, but an elevated level of matrix metalloproteinases (especially type 9) in the inferior tear meniscus is believed to be responsible for stromal thinning and perforation.^5^ There is very little information in the literature about corneal perforation in patients with ocular rosacea.^27,28,29^ In one of the reported cases keratoplasty was performed to repair an area of perforation extending to the sclera; in other cases, amniotic membrane transplantation was performed after the application of cyanoacrylate tissue adhesive and bandage contact lenses proved insufficient.^27,28,29^ In one of our two patients who developed this complication, tissue adhesive and amniotic membrane transplantation were used to repair the perforation and achieve re-epithelization; in the other, only tissue adhesive and bandage contact lens were used. These results suggest that tissue adhesive and, if necessary, amniotic membrane transplantation are effective in the treatment of perforations due to ocular rosacea.

Due to the retrospective nature of this study, it has certain limitations. As there were no controls for the treatment approaches used, it is difficult to clearly determine the effect of each drug or treatment.

## CONCLUSION

Ocular rosacea may affect the ocular surface to varying degrees and can threaten patients’ vision, but in most cases ocular surface inflammation can be managed with appropriate medical treatment. It is important to choose treatment approaches according to the patients’ clinical characteristics and to follow these patients closely.

## Ethics

Ethics Committee Approval: It was taken, Informed Consent: It was taken, Peer-review: Externally peer-reviewed.

## Figures and Tables

**Table 1:**
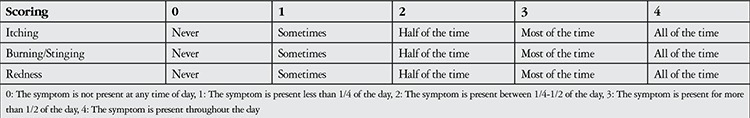
Scoring system of the patients’ ocular symptoms

**Table 2:**
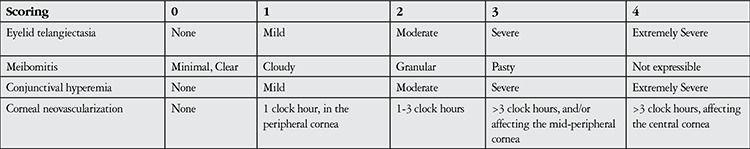
Scoring system of the patients’ ocular signs

**Table 3:**
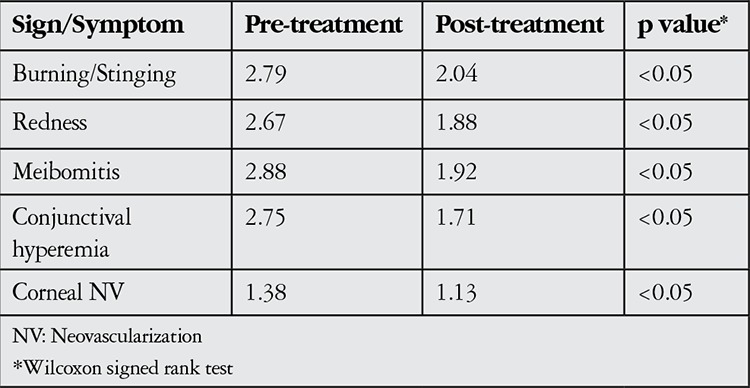
Comparison of total pre- and post-treatment scores of patients’ signs and symptoms
